# The Repetition of Prescriptions

**Published:** 1914-07-25

**Authors:** 


### The Repetition of Prescriptions.

It is evident from the nature of certain inquiries that have been addressed by the Foreign Office to the British Medical Association and the Pharmaceutical Society that the Government has under consideration the question of the desirability of amending the law regulating the sale of poisons in such a way as to place some limit on the number of times a prescription for morphine, cocaine, and other narcotic drugs may be dispensed. There can be little doubt that danger often arises as a result of the absence of any restriction on the repetition of prescriptions of this kind—a danger which is avoided in hospitals and similar institutions where medicines cannot be repeated indefinitely. The Government, therefore, is considering whether it will not be practicable and advisable to embody regulations governing this matter in the legislation which will have to be framed to give effect to the provisions of the International Opium Convention. The British Medical Association has advised the Foreign Office that legislation should be introduced to provide (1) that medical practitioners should state in their prescriptions the number of times for which these are to be used; and (2) that the compounder should under penalty be bound to comply with the limitation. The Association has long recognised the dangers arising from the ease with which persons in this country can obtain powerful drugs by means of a doctor's prescriptions, and as this danger can be avoided with a minimum of inconvenience there seems to be no reason why the suggested legislation should not be enacted.

